# Novel Loss of Function Variant in *SOST* From Chinese Family Results in Sclerosteosis 1

**DOI:** 10.1002/mgg3.70109

**Published:** 2025-07-02

**Authors:** Yufan Guo, Xintao Wu, Yuting Jin, Yu Gu, Yuting Lou, Pu Miao, Ye Wang, Bijun Zhang, Xueting Lin, Chudi Zhang, Jianhua Feng

**Affiliations:** ^1^ Department of Pediatrics The Second Affiliated Hospital of Zhejiang University School of Medicine Hangzhou China; ^2^ Zhejiang University School of Medicine Hangzhou China

**Keywords:** abnormal facial expressions, facial numbness, genetic, sclerosteosis 1, *SOST*

## Abstract

**Background:**

*SOST* encodes a secreted glycoprotein that is similar in sequence to the differential screening‐selected gene aberrative in neuroblastoma (DAN) family of bone morphogenetic protein (BMP) antagonists. Pathogenic variants in the *SOST* gene result in sclerosteosis, van Buchem disease (VBD), or craniodiaphyseal dysplasia. *SOST*‐related genetic disorders are very rare, and limited studies have reported variants associated with sclerosteosis.

**Methods:**

Clinical tests such as magnetic resonance imaging (MRI), computed tomography (CT), emission computed tomography (ECT), electromyogram (EMG), routine blood tests, and physical examinations were conducted for the proband. Trio‐whole exome sequencing (Trio‐WES) was performed, and the rare variants (allele frequency < 0.01) in the exon and splicing regions were selected for further pathogenic evaluation. Candidate pathogenic variants were validated through Sanger sequencing. The wild and mutant *SOST* sequences were cloned into the pcDNA3.1 expression vector, and the RNA and protein expression levels were investigated in the HEK293T cell line.

**Results:**

In this study, we present a case study of a proband who displays abnormal facial expressions accompanied by numbness. The results of the brain MRI show thickening of the skull and disappearance of the diplopia signal. The temporal bone CT scan indicates diffuse osteosclerosis affecting the bilateral ossicular chains and internal auditory meatus, as well as stenosis of the bilateral internal auditory meatus. Trio‐WES sequencing detected a novel homozygous variant in the proband: NM_025237.3(*SOST*): c.327C>A (p.Cys109*), which was also validated in his sister from the same family. According to the ACMG guidelines, the variant is classified as “likely pathogenic.” The in vitro experiments demonstrated that the variant caused a decrease in *SOST* expression at RNA and protein level and produced a truncated protein.

**Conclusion:**

The report presents new evidence for the clinical diagnosis of *SOST*‐related facial numbness and expands the variant spectrum of *SOST*.

## Graphical abstract

In this study, we present a case study of a proband who displays abnormal facial expressions accompanied by numbness. The results of the brain MRI show thickening of the skull and disappearance of the diplopia signal. The temporal bone CT scan indicates diffuse osteosclerosis affecting the bilateral ossicular chains and internal auditory meatus, as well as stenosis of the bilateral internal auditory meatus. Trio‐WES sequencing detected a novel homozygous variant in the proband: NM_025237.3(*SOST*): c.327C>A (p.Cys109*), which was also validated in his sister from the same family. According to the ACMG guidelines, the variant is classified as “likely pathogenic.” The in vitro experiments demonstrated that the variant caused a decrease in *SOST* expression at the RNA and protein level and produced a truncated protein.

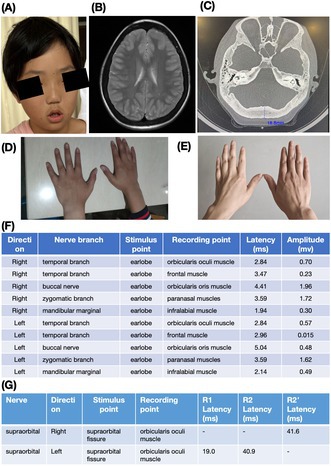

## 1 Introduction

Sclerostin, encoded by the sclerostin (*SOST*, OMIM: 605740) gene, is a 190‐amino acid long secreted glycoprotein of the DAN family which is highly conserved across vertebrate species (Sebastian and Loots 2017; Omran et al. 2022). Sclerostin was identified as a key regulator of bone homeostasis, modulating bone formation by osteoblasts through inhibition of the canonical Wnt signaling (Moretti and Iolascon 2023). The *SOST* gene expressed in osteocytes is highly conserved across vertebrate species. The gene located on chromosome 17q12‐21 was described in the pathogenesis of three disease: sclerosteosis 1 (SOST1, OMIM: 269500), craniodiaphyseal dysplasia, autosomal dominant (CDD, OMIM: 122860) and van Buchem disease (VBD, OMIM: 239100) (Moretti and Iolascon 2023; Sebastian and Loots 2018). All three diseases are related to the *SOST* gene, but different in their patterns of inheritance and pathogenic mechanisms.

CDD is a severe form of bone dysplasia characterized by extensive hyperostosis and sclerosis throughout the body, with significant involvement of the skull and facial bones. CDD is caused by heterozygous variants in the *SOST* gene (Kim et al. 2011). SOST1 and VBD are less severe autosomal recessive inheritance diseases. Patients with them exhibit a progressive and generalized skeletal overgrowth and sclerosis on radiographic examination. Additionally, there is a general enlargement with cortical thickening and increased density observed in the ribs, clavicles, pelvis, diaphysis of long bones, and the tubular bones of the hands and feet. Thickening of the skull can result in various clinical outcomes, including deafness, visual disturbances, facial nerve palsy, and neurological pain due to narrowing of the cranial foramina (Sebastian and Loots 2018; Tanaka and Matsumoto 2021). SOST1 has been linked to loss of function (LOF) mechanism (Ekhzaimy et al. 2022) and is caused by homozygous/compound heterozygous variants in the *SOST* gene. VBD is caused by a 52‐kb deletion approximately 35 kb downstream of the *SOST* gene, which leads to a SOST‐specific regulatory element missing (Balemans et al. 2002).

To date, only a few pathogenic variants have been identified in *SOST*‐related patients. In the present study, we report a sclerosteosis 1 proband with a novel homozygous variant that results in LOF in the Sclerostin locus. This study would expand the variant spectrum of *SOST* and provide new evidence for the clinical diagnosis of *SOST*‐related diseases.

## 2 Materials and Methods

### 2.1 Patients and Ethnic Approval

This study was approved by the Institutional Ethics Board of the Second Affiliated Hospital Zhejiang University School of Medicine (no. JXSETYY‐YXKY‐20210053), and informed consent was obtained from the patient's parents. Magnetic resonance imaging (MRI), computed tomography (CT), emission computed tomography (ECT), electromyogram (EMG), routine blood tests, and physical examinations were conducted. The procedures were performed in accordance with the Declaration of Helsinki (2013 revision).

### 2.2 Genetic Testing

Blood samples were collected from the individuals and his family. Genomic DNA was extracted from the blood samples and purified using the TIANamp Blood DNA Kit (DP348‐02, TIANGEN, China) according to the manufacturer's instructions. Following purification, 1 μg of genomic DNA per sample was fragmented, purified again, and captured using Berry's NanoWES Human Exome V1.0 (Berry Genomics, Beijing, China) according to the manufacturer's protocol. The libraries were sequenced on the NovaSeq 6000 Sequencing Platform (Illumina, USA). The raw data was converted to fastq format data using bcl2fastq (https://github.com/savytskanatalia/bcl2fastq). After removing low‐quality reads and adaptors using TrimGalore (https://github.com/FelixKrueger/TrimGalore), the clean paired‐end reads were aligned to the human reference genome (GRCh38/hg38) using Burrows‐Wheeler Aligner (BWA) (Li and Durbin 2009). Variant calling was performed using Verita Trekker Variants Detection System by Berry Genomics and the third‐party software GATK (https://software.broadinstitute.org/gatk/) (Van der Auwera et al. 2013). ANNOVAR and the Enliven Variants Annotation Interpretation System authorized by Berry Genomics were used by variant annotation and interpretation (Wang et al. 2010). The pathogenicity of the filtered variants was classified according to the ACMG guidelines (Richards et al. 2015). Candidate pathogenic variants were confirmed by Sanger sequencing in this family.

### 2.3 Sanger Sequencing

The candidate variant was performed on the proband, the other family members, and the healthy control sample. The primers were designed by Primer 5. PCR conditions: initial denaturation at 95°C for 4 min, followed by 35 cycles at 95°C for 20 s, 56°C for 20 s, and 72°C for 20 s. PCR products were purified and sequenced using an ABI 3730XL (Applied Biosystems, Foster, CA, USA). The data from Sanger sequencing was analyzed by SnapGene software according to the sequences of the *SOST* gene (NM_025237.3).

### 2.4 Protein Structure Analysis

The solved three‐dimensional (3D) crystal structure of Sclerostin was used for analysis of the possible effects of identified variants on the protein. Possible effects of protein‐altering variants (PAVs) were predicted on the basis of the wild‐type amino acid position and interactions with other amino acids, other proteins, or ligands, and biophysical differences with the mutant amino acid, similarly as described previously (Balemans et al. 1999). A clustering of heterozygous missense variants in the crucial chromatin modifier WDR5 defines a new neurodevelopmental disorder. The protein structure used (PDB: 6l6r) contains two copies of Sclerostin and two copies of low‐density lipoprotein receptor‐related protein 6. The predicted three‐dimensional structures of the protein models were visualized using PyMOL (Version 2.5; Schrödinger LLC).

### 2.5 Plasmid Construction and Transfection

The full‐length *SOST* cDNA sequence was cloned into the phage expression vector (Bioeagle Biotech Company Ltd., Wuhan, China). Restriction sites and full‐length *SOST* were inserted into the phage plasmid with primers (phage‐*SOST*‐SalI‐F/phage‐*SOST*‐NotI‐R) to construct the recombinant vector phage‐*SOST*‐wt (wild type). A pair of mutant primers, *SOST*‐mut‐F and *SOST*‐mut‐R, were designed to amplify the fragment 2. Fragment 1 was amplified by using phage‐*SOST*‐SalI‐F and *SOST*‐mut‐R as primers, as listed in Table 1. Using a 1:1 mixture of Fragment 1 and Fragment 2 as a template, fragment phage‐*SOST*‐mut containing the mutation c.327C>A, p.Cys109* was obtained by overlap extension PCR using phage‐*SOST*‐SalI‐F and phage‐*SOST*‐NotI‐R as primers (Table 1). The two recombination plasmids mentioned above were confirmed by sequencing. The human embryonic kidney 293T cells were cultured in DMEM supplemented with 10% fetal bovine serum (Gibco, Grand Island, NY, USA) and incubated at 37°C and 5% CO_2_ atmosphere. The 293T cells were transiently transformed with phage‐SOST‐wt/mut plasmid using Lipofectamine 2000 (Invitrogen, Carlsbad, CA, USA) following the manufacturer's instructions. The cells were transfected and cultured for 48 h, and then total RNA and proteins were extracted and verified by real‐time‐PCR (qPCR) and western blotting.

**TABLE 1:** The primers used in the construction of the plasmid, point mutation, and qPCR.

Primer name	Sequences
phage‐*SOST*‐SalI‐F	TGACGTCGACAATGCAGCTCCCACTGGCCC
phage‐*SOST*‐NotI‐R	CGACGCGGCCGCCTAGTAGGCGTTCTCCAG
pcDNA3.1‐*SOST*‐EcoRI‐F	ggtggaattcAATGCAGCTCCCACTGGCCC
pcDNA3.1‐*SOST*‐Xbal‐R	gccctctagaCTAGTAGGCGTTCTCCAGCT
*SOST*‐mut‐F	TGCTCCGGCCAGTGAGGCCCGGCGCGCCTG
*SOST*‐mut‐R	CAGGCGCGCCGGGCCtCACTGGCCGGAGCA
*SOST*‐QPCR‐F	TGCTGGTACACACAGCCTTC
*SOST*‐QPCR‐R	ACTCGGACACGTCTTTGGTC

### 2.6 Real‐Time PCR (RT‐PCR)

RT‐PCR was used to detect the expression of *SOST* mRNA. Briefly, 293T cells were transiently transformed with phage‐*SOST*‐wt/mut plasmids, and the RNA of the cells was extracted following the manufacturer's protocol (Takara, Japan). The primer sequences are listed in Table 1 (*SOST*‐QPCR‐F/R).

### 2.7 Western Blotting

The proteins were collected and quantified using the BSA reagent (Thermo Fisher Scientific, Waltham, MA, USA). Subsequently, they were resolved on a 10% polyacrylamide gel containing a sodium dodecyl sulfate, transferred onto a polyvinylidene fluoride membrane (Millipore, Bedford, MA, USA), and incubated overnight at 4°C with primary antibodies (1:1000 dilution) against *SOST* (Absin, Shanghai, China) and GAPDH (Cell Signaling Technology, Danvers, MA, USA). After washing, the blots were visualized using a chemiluminescent substrate and then analyzed by ImageJ software. To support the conclusion drawn in this study, another pcDNA3.1 vector was conducted and performed in 293T cells by using the same experimental method as above. The primer sequences are listed in Table 1.

### 2.8 Statistical Analysis

The results are presented as the mean ± SEM. Statistical analysis was conducted using SPSS software, version 21.0 (SPSS Inc., Chicago, IL, USA). Comparisons between groups were made using one‐way ANOVA. Paired data were assessed using a two‐tailed Student's *t*‐test. Statistical significance was considered when *p* < 0.05.

## 3 Result

### 3.1 Clinical Description

**FIGURE 1:**
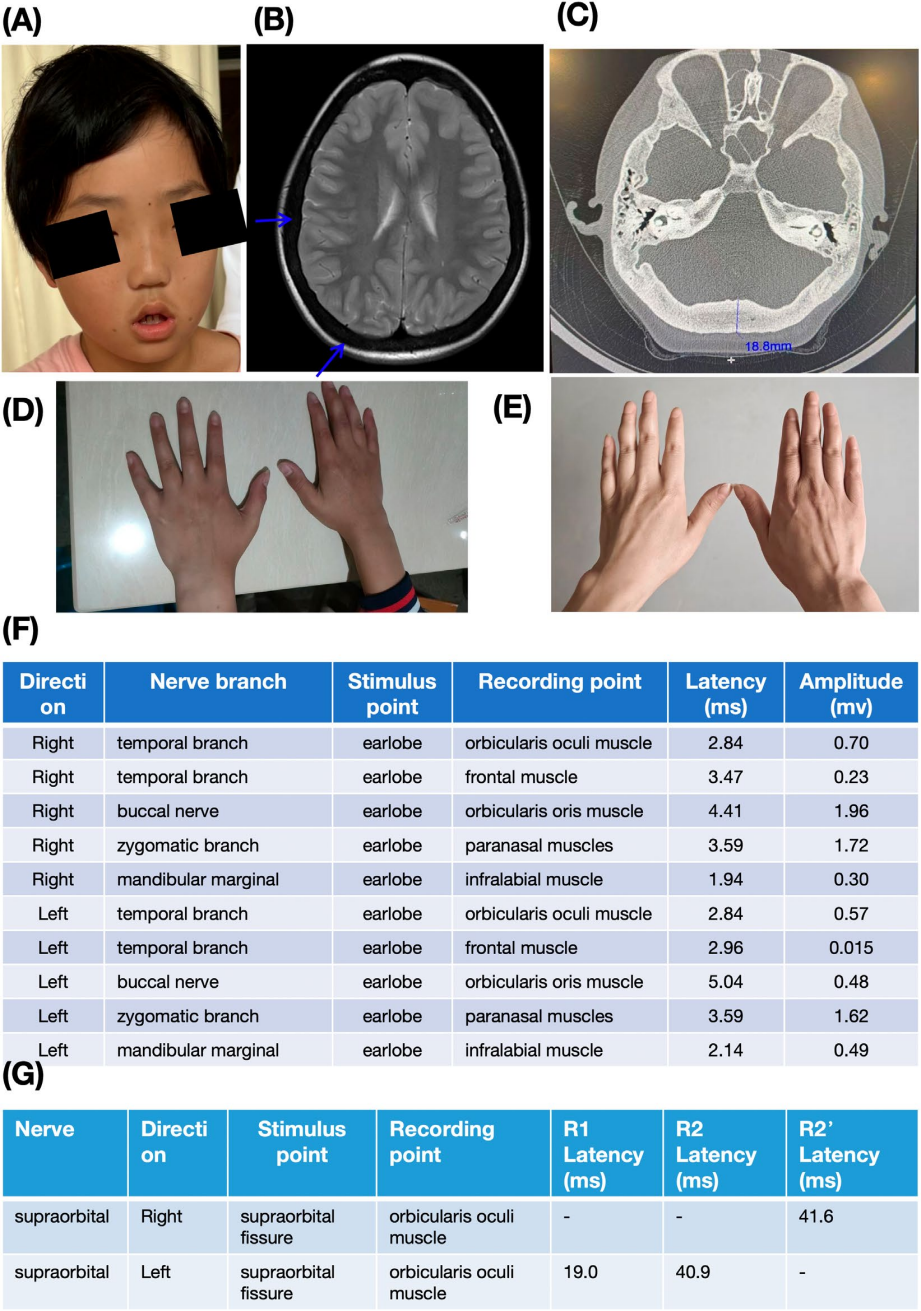
Clinical features of *SOST* genetic variant. (A) Facial photographs of the proband who exhibited abnormal facial expressions with numbness. (B) The brain MRI indicates that the skull thickens and the signal of the diplopia disappears (blue arrow). (C) Temporal bone CT results indicate diffuse osteosclerosis, involving bilateral ossicular chains and internal auditory meatus, and bilateral internal auditory meatus stenosis. (D) The hands of the proband. (E) The hands of the sister. (F) Measurement of motor afferent function of the facial nerve. (G) Measurement of blink reflex.

During the physical examination, it was observed that the patient's bilateral forehead lines had disappeared, the distance between the eyes had widened, the closure of both eyes was incomplete, the bilateral nasolabial sulcus had become shallow, the nasal bridge was low, the mouth angle was slightly tilted to the left, and there was tenderness in the bilateral maxillary sinus area (Figure 1A). The biochemical examination shows elevated levels of alkaline phosphatase (504 U/L, reference: < 300 U/L), glycylproline aminopeptidase (175 U/L, reference: 38–116 U/L), phosphorus (2.01 mmol/L, reference: 0.95–1.95), osteocalcin (> 300.0 μg/L), β collagen degradation product (4892.0 ng/L), and total type I collagen amino terminal peptide (> 1200.0 μg/L).

The facial nerve MRI results show that the bilateral internal auditory meatus is narrow and the facial auditory nerve is slender and unclear. The brain MRI indicates that the skull thickens and the signal of the diplopia disappears, bilateral mastoiditis, adenoid hypertrophy, paranasal sinusitis, and mastoiditis at both middle ear (Figure 1B). Temporal bone CT findings indicate diffuse osteosclerosis, involving bilateral ossicular chains and internal auditory meatus, and bilateral internal auditory meatus stenosis (Figure 1C). Deviation of terminal phalanges in the proband and his sister (Figure 1D,F). The bending of my sister's fingers is more noticeable. Bone ECT examination indicates that there is no obvious abnormality in the whole‐body bone. The EMG examination results show prolonged latency of motor conduction in the left and right facial nerves, including the orbicularis oculi, orbicularis oris, and paranasal muscles (Figure 1F). The amplitude of motor conduction evoked potentials decreased in the left facial nerve (at the orbicularis oculi muscle, frontal muscle, orbicularis oris muscle, and infralabial muscle) and the right facial nerve (at the orbicularis oculi muscle, frontal muscle, and infralabial muscle). In measuring the blink reflex, it was found that the R1 and R2 waves were not elicited and the latency of R2′ wave was prolonged with right side stimulation. Similarly, left side stimulation resulted in prolonged latency of R1 and R2 waves, and R2′ waves were not elicited (Figure 1G). These above results suggest bilateral neuropathy.

### 3.2 Genetic Screening and Analysis of SOST

**FIGURE 2:**
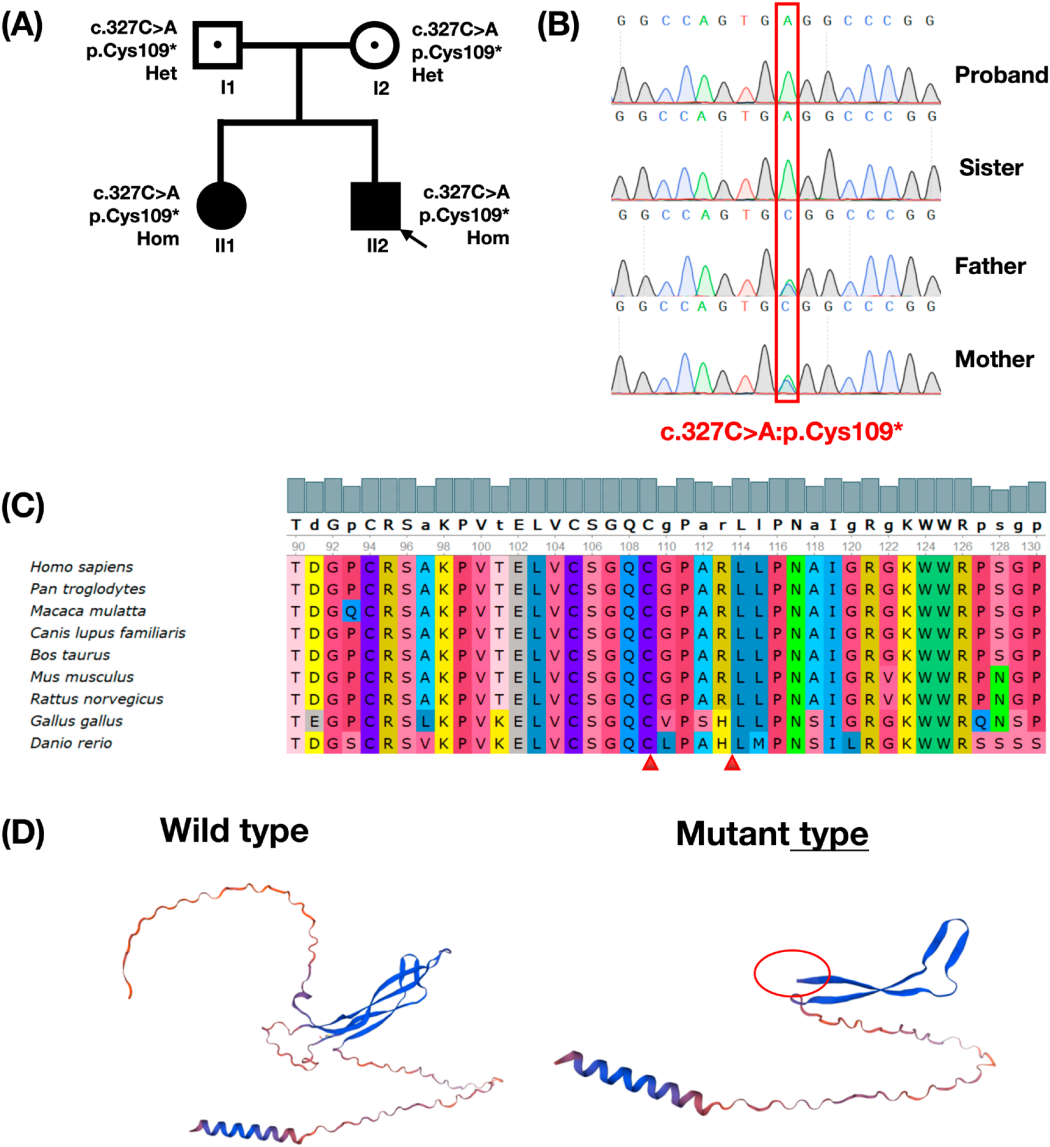
Genetic screening and analysis of *SOST*. (A) Two‐generation pedigree of the family showing two affected siblings (NM_025237.3). (B) Sequencing results showed that mutation p.Cys109* were successfully introduced. (C) Conservativeness analysis of *SOST* variant loci. (D) Prediction of the 3D protein structure of the *SOST* gene.

We identified a homozygous variant in *SOST* [NM_025237.3: c.327C>A, (p.Cys109*)] in the proband (variant/total depth: proband [153/153], father [90/178], mother [85/163]); this variant was validated by Sanger sequencing (Figure 2A,B). The sister with the similar phenotype also carried the same homozygous variant. It is a nonsense variant that results in an amino acid change from Cysteine to a stop codon at the 109th residue of *SOST* (Figure 2C). The variant is not collected in public databases such as gnomAD and is classified as likely pathogenic (LP) according to ACMG guidelines (LP evidence: PVS1_Strong: p.Cys109* is a nonsense mutation, resulting in LOF and causing the associated disease) (Figure 2D). The transcript (NM_025237) is the only one transcript for *SOST* and is biologically significant. The termination codon caused by p.Cys109* locates at the last 50 bp of the penultimate coding exon. It is predicted that this mutation may not activate nonsense‐mediated mRNA degradation, leading to a protein sequence deletion of greater than 10%. PM2_supporting: p.Cys109* is not collected in public databases such as gnomAD; PM3: The disease is an autosomal recessive inheritance. There were two homozygous cases (inherited from the heterozygous parents).

### 3.3 Evaluation the Novel SOST Variant on Gene Expression

**FIGURE 3:**
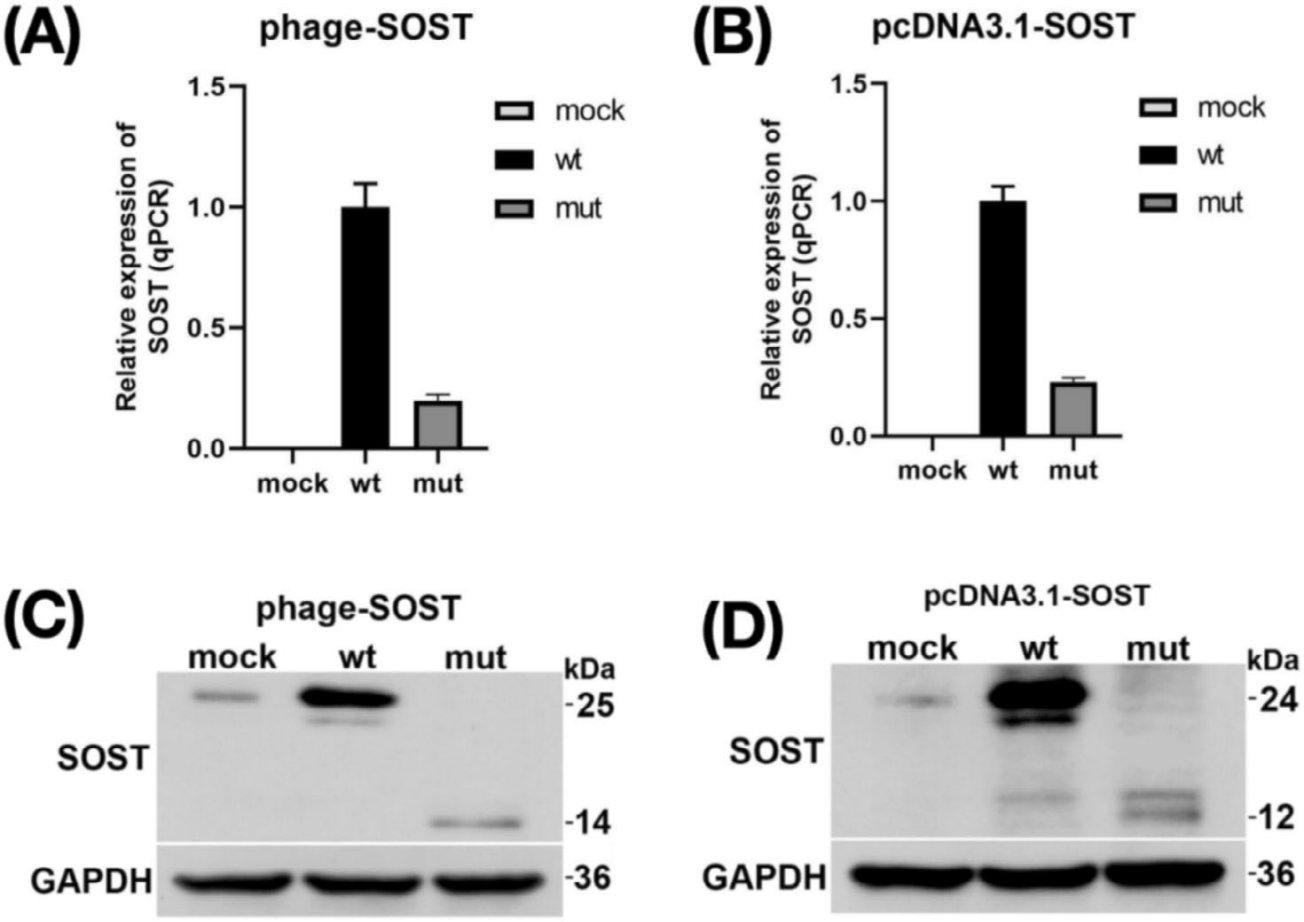
Evaluation of the effect of p.Cys109* on *SOST* by in vitro experiments. (A, B) RNA expression analysis of *SOST* by qPCR between the variant (p.Cys109*) and the wild‐type. Expression levels were normalized to those of the ACTB control. Three technical replicates were performed for each treatment group. (C, D) Evaluation of wild‐type and p.Cys109* *SOST* variant protein levels by western blot.

To assess the impact of the p.Cys109* variant on *SOST* expression in cells, we created both *SOST* wild‐type and mutant expression vectors. The mutant vector was generated using site‐directed mutagenesis to introduce the p.Cys109* variant. Upon transfection of these constructs into HEK293T cells, we observed a significant reduction in mRNA levels of the mutant type compared to the wild type (Figure 3A). The expression from the above constructs was further confirmed by another pcDNA3.1 vector (Figure 3B). Western blotting (WB) results showed that compared to the wild type *SOST* (25 kDa, Phage‐*SOST*‐wt), the mutant type produced a truncated peptide chain leading to premature termination (14 kDa, phage‐*SOST*‐mut). The protein expression of the variant is significantly reduced (Figure 3C). The protein expression from the above constructs was further confirmed by another pcDNA3.1 vector (Figure 3D).

## 4 Discussion

Sclerostin, secreted by osteocytes, is a bone tissue‐specific inhibitor of the Wnt/β‐catenin pathway. It negatively regulates osteogenic differentiation and bone formation while promoting osteoclastogenesis and bone resorption (Marini et al. 2023). Wnt signaling regulates both skeletal development and bone homeostasis. Variants that result in LOF or gain of function in various members of the Wnt pathway have been demonstrated to lead to severe skeletal abnormalities (Sebastian and Loots 2018). Osteoporosis is a systemic skeletal disorder marked by diminished osteoblast differentiation, primarily due to the overexpression of the SOST gene (Niveria et al. 2024). It is known that the downregulation of the *SOST* gene may lead to SOST1 and VBD diseases. VBD patients differ from those with sclerosteosis in that they do not display syndactyly or nail dysplasia and maintain a normal stature (van Lierop et al. 2013). In our research, we found p.Cys109* in the *SOST* gene leads to a decrease in protein expression through in vitro experiments. The proband displays abnormal facial expressions and numbness, but no other clinical features are evident. Brain MRI further reveals thickening of the skull, while temporal bone CT shows diffuse osteosclerosis. The proband has normal stature and no syndactyly. The patient's phenotype is relatively mild, which can be somewhat confusing in the early stages of clinical diagnosis, and gene testing helps to confirm the diagnosis.

The pathogenic region for SOST1 and VBD was located on chromosomes 17q12‐q21 due to clinical and radiological similarities. Evidence from 11 VBD patients from the Netherlands and 2 close relatives with sclerosis supported this finding (Balemans et al. 1999). Two independent studies then identified the *SOST* gene, which mutates in patients with sclerosing ossification, through homozygous mapping and positional cloning of families affected by sclerosing ossification (Brunkow et al. 2001; Balemans et al. 2001). However, no variants were detected in the *SOST* coding region in VBD patients. Instead, a 52 kb deletion that occurred in the *SOST*‐MEOX1 intergenic region on 17q12‐q21 was identified as the pathogenic factor in VBD patients (Staehling‐Hampton et al. 2002).

To date, only a limited number of *SOST* variants have been reported associated with SOST1 or VBD. In addition to the initially reported 52 kb deletion on 17q12‐q21, another homozygous splicing variant has been reported (Balemans et al. 2005). In the present study, we describe a Chinese patient with a novel mutation in the *SOST* gene. From a mechanistic perspective, both variants lead to a decrease in *SOST* protein expression (Van der Auwera et al. 2013; Wang et al. 2010). In order to investigate the homozygous variant (NM_025237.3: c.327C>A, p.Cys109*) detected in our proband, we conducted in vitro experiments which demonstrated that the variant influenced the protein expression of *SOST*. This function is similar to reported variants. Therefore, we speculate that this variant is the cause of the patient's illness. However, in future studies, further researchers should use mouse or zebrafish as models to confirm the mechanism of *SOST*‐induced VBD.

## 5 Conclusion

In this study, we report a sclerosteosis 1 proband with a novel homozygous variant which results in loss function of *SOST*. Our report expands the variant spectrum of *SOST* gene and provides new evidence for the clinical diagnosis of *SOST*‐related VBD.

## Author Contributions

Y.G. and B.Z. contributed to the design of the study and draft the manuscript; Y.L., Y.J., and X.W. contributed to analyze the data; P.M., Y.W., Y.G., X.L., and C.Z. contributed to recruit study participants and collect samples; and J.F. contributed to the design of the study and revise the manuscript. All authors read the final version of the manuscript.

## Ethics Statement

All procedures performed in this study involving human participants were in accordance with the Declaration of Helsinki (as revised in 2013). This study was reviewed and approved by the institutional review board of the Second Affiliated Hospital Zhejiang University School of Medicine (approval number is No. 20221005).

## Consent

Written informed consent was obtained from the parent/legal guardian of the patient for publication of the details of their medical case and any accompanying images.

## Conflicts of Interest

The authors declare no conflicts of interest.

## Data Availability

The data that support the findings of this study are openly available in ClinVar database at https://www.ncbi.nlm.nih.gov/clinvar/variation/2583091/?oq=SCV004041808&m=NM_025237.3(SOST):c.327C%3EA%20(p.Cys109Ter), reference number SCV004041808.
